# Evaluation of fluorescent in-situ hybridization technique for diagnosis of malaria in Ahero Sub-County hospital, Kenya

**DOI:** 10.1186/s12879-017-2875-x

**Published:** 2018-01-08

**Authors:** Regina Kandie, Rachel Ochola, Kariuki Njaanake

**Affiliations:** 1 Department of Medical Microbiology, College of Health Sciences, University of Nairobi, Nairobi, Kenya; 2 National Malaria Control Program, Ministry of Health, Nairobi, Kenya; 3 Technical University of Kenya, Nairobi, Kenya

**Keywords:** Malaria, Fluorescent in-situ hybridization, Microscopy, Rapid diagnostic tests, Sensitivity, Specificity

## Abstract

**Background:**

Malaria is a major cause of morbidity and mortality. Treatment of malaria in a timely manner could avert deaths. Treatment ultimately relies on the rapid and accurate diagnosis. Fluorescence in situ hybridization (FISH), a cytogenetic technique based on detection of specific nucleic acid, has the potential to address the limitations of the current diagnostic approaches. This study investigates further the performance of FISH for the diagnosis of malaria in a rural setting in Western Kenya.

**Methods:**

Blood samples from 302 patients presenting with fever (temperature ≥ 37.5 °C) were examined for malaria using the Giemsa microscopy (GM), rapid diagnostic test (RDT), polymerase chain reaction (PCR) and FISH.

**Results:**

The sensitivity and specificity of FISH was 85.6% and 96.2% respectively, while the corresponding values for GM were 82.2% and 100% respectively. RDT and PCR had sensitivities of 91.1% and 98.9%, respectively with their specificities being 89.6 and 100%, respectively. The positive predictive values for RDT, GM, FISH and PCR were 78.8%, 100%, 90.6% and 100%, respectively. The negative predictive values for RDT, GM, FISH and PCR were 96.0%, 93.0%, 94.0% and 99.5%, respectively. Their respective diagnostic accuracies were 90.1%, 94.7% 93.0% and 99.7%.

**Conclusion:**

The present study demonstrates that the specificity and reproducibility of FISH assays are high, thus adding to the growing evidence on the potential of the technique as an effective tool for the detection of malaria parasites in remote settings.

## Background

Malaria is one of the most important public health problems worldwide, and a leading cause of morbidity and mortality in many developing tropical countries, with young children and pregnant women being the most affected groups within populations [1]. An estimated 3.4 billion people in 106 countries and territories live in high malaria risk areas [2]. Worldwide, a child dies of malaria every 2 min which translates to about 3000 children every day [3]. About 88% of all malaria cases in 2015 are estimated to have occurred in sub-Saharan Africa where it is still the main cause of death and a major threat to child health, followed by the World Health Organization (WHO) South-East Asia Region (7%) and the WHO Eastern Mediterranean Region (2%). During the same year, it was estimated that most (92%) malaria-related deaths were in the WHO African Region, followed by the WHO South-East Asia Region (6%) and the WHO Eastern Mediterranean Region (2%) [4]. Malaria is mainly concentrated in low and middle-income countries where it mostly affects the poorest and marginalized communities. Such communities have the highest risks associated with malaria, and the least access to effective services for prevention, diagnosis and treatment [4, 5].

In Kenya, about 52% of the population is at risk of contracting malaria [6]. The goal of the National Malaria Strategy 2009–2017 is to reduce morbidity and mortality associated with malaria by 30% by the year 2009 and to maintain it through 2015 [7]. Countrywide, prevalence of malaria has declined from 11% to 8% between 2010 and 2015. The endemic region along Lake Victoria has the highest malaria prevalence (27%) [7, 8].

Following WHO’s recommendation that all cases of suspected malaria be confirmed using parasite-based diagnostic testing, either microscopy or rapid diagnostic test (RDT) before commencement of treatment, there has been an impressive increase in the proportion of suspected malaria cases receiving a diagnostic test since 2010, especially in the African Region [8]. However, malaria diagnosis through microscopy has been shown to have low sensitivity and specificity, is laborious and requires highly trained personnel which negatively affects health outcomes and optimal use of resources [9–11].

Malaria RDTs provide a valuable tool especially in field studies as they are able to counter the inadequacies of other diagnostic approaches. In countries where malaria is endemic, they provide an alternative approach in settings where microscopy is not feasible [12]. Moreover, they can be used to complement microscopy as they provide timely results and in settings where microscopy experience is limited as is the case in most non-endemic countries [12, 13]. The method is based on the immunochromatographic principles, that is, chromatography coupled with monoclonal antibodies that capture antigens of the parasite including pan-lactate dehydrogenase (pLDH) or histidine-rich protein-2 (HRP-2) [14]. As with microscopy, RDTs have limited detection threshold, particularly in situations where parasitaemia is low [14, 15]. Moreover, despite its wide use, the RDT method based on serum antibody is also not specific [16, 17].

Currently, polymerase chain reaction (PCR) assay is used chiefly for research purposes or for speciation in reference laboratories. This method involves detecting circulating malaria parasites by detecting parasite DNA through amplification of ribosomal RNA genes. This can detect parasites at low parasitaemia, even below 5 parasites/μl of blood, for all five human *Plasmodium spp.* [18]. However, molecular techniques are expensive and require specialized laboratory facilities and highly trained personnel, owing to the related cost of labor and difficulties in accessing the reagents, compared to the examination of blood smears [19]. These drawbacks present an important impediment to its adoption in malaria reference laboratories located in endemic resource-poor regions of the world.

In Kenya, Giemsa microscopy (GM) is traditionally the gold standard for diagnosis. Under optimum conditions, microscopy can detect 20–50 parasites per μL blood, but such sensitivity is rarely achieved in routine diagnosis. On the other hand, parasite nucleic acids can be detected using PCR. Although this technique may be slightly more sensitive than smear microscopy, it is of limited utility for the diagnosis of acutely ill patients in the standard healthcare setting due to its unavailability and long turnaround time to results. PCR results are often not available quickly enough to be of value in establishing the diagnosis of malaria in such patients. Other alternative laboratory methods for the detection of malaria parasites including the quantitative buffy-coat centrifugal haematology system, immunofluorescence and ELISA tests for the detection of *P. falciparum* antigen have been recently developed. None of these tests however, are used routinely as they are far too complicated or expensive for use in routine diagnosis especially in a developing countries including Kenya. There is therefore a need for sensitive, easily accessible and affordable diagnostic tools to facilitate targeted treatment and management of malaria. In addition, new diagnostics that can detect low levels of parasitaemia in asymptomatic individuals and, in the case of *P. vivax*, in the blood of asymptomatic individuals who may serve as reservoirs for mosquito infections, are much needed [20].

One of the promising molecular techniques in malaria diagnosis is the fluorescent in-situ hybridization (FISH) assay. This is a cytogenetic technique that is able to detect and localize the specific nucleic acid (DNA or RNA) sequences by hybridizing with complementary sequences labeled with fluorescent probes. The assay gives results within 45 mins and can circumvent the drawbacks characterising PCR such as requirement of sophiticated equipment, highly trained personnel and prolonged time to results. Specific FISH assays that can be deployed in peripheral laboratories of limited resource nations where poverty-related diseases including malaria and tuberculosis are endemic, have been developed [21, 22]. There is accumulating evidence that FISH assay technique can be a valuable tool for the diagnosis of malaria under routine clinical settings. Indeed, a recent study done in a malaria holoendemic region of Kenya showed that P-Genus FISH assay has great potential in detecting low density parasites [23]. This study aimed at generating additional evidence on use of FISH in a clinical setting by evaluating the performance of the technique in malaria diagnosis in a rural malaria-endemic community in Western Kenya.

## Methods

### Study area

The study was conducted in Ahero sub-County Hospital, Kisumu County of former Nyanza province, Western Kenya. The County covers an area of 2085.9 km^2^ and has a population of 968,909 [20]. This area is situated in the lake region malaria epidemiological zone. The massive vegetation cover, swampy low altitude and consistent rainfall make the region highly conducive for mosquito breeding and high malaria transmissions.

### Study design

This was a descriptive cross-sectional study in which blood samples were collected from patients presenting with fever of ≥37.5 °C and examined for malaria parasites using rapid diagnostic test (RDT) kit, Giemsa microscopy (GM), polymerase chain reaction (PCR) and fluorescent in-situ hybridization (FISH).

### Sample size

The minimum required sample size for the study was calculated using the formula by Lwanga and Lemeshow [24].

$$ n={z_{\propto}}^2\frac{p\left(1-p\right)}{d^2} $$

Where;

*n* is the sample size

*z*_∝/2_ is the normal standard deviate for a given level of significance (5%, *z*_∝/2_ = 1.962)

*p* is the prevalence of infection expressed as a proportion

*d* is the desired level of precision (0.05).

Malaria prevalence around Lake Victoria is estimated to be 26.7% [25]. Using 5% as the precision level and level of significance, this gave a minimum sample size (n) of 302 patients.

### Sampling procedure

Patients visiting the outpatient clinics of Ahero sub-County Hospital in October 2016 and presenting with fever (≥ 37.5 °C) and with suspected malaria were enrolled sequentially into the study after informed consent was obtained. Exclusion criteria were lack of consent from the caregivers and symptoms associated with signs of severe malaria as defined by WHO [26]. Also excluded were those with had been on anti-malarial drugs within the previous 7 days.

### Laboratory procedures: Collection and examination of blood samples

From each enrolled subject, 2 ml of venous blood sample was drawn and examined for the presence of malaria parasites using the GM, RDT, PCR and FISH.

#### Giemsa microscopy

Giemsa microscopy was performed as outlined in the WHO guidelines [27]. Blood films (thin and thick) were prepared and then stained using 10% Giemsa stain. The slides were examined microscopically using ×1000 magnification. Thick blood film were examined for the presence of parasites and density while species confirmation was performed on thin film. Quantification of malaria parasites was done for positive slides by counting the parasites against 200 white blood cells. Samples were classified as negative if no malaria parasites were observed following examination of 100 microscopic fields. The readings were done by two independent microscopist who were blinded. In cases where the results were discordant, the readings from a third microscopist served as a tie breaker.

#### Rapid diagnostic tests

Blood samples were tested for malaria parasite antigen using SD Bioline Malaria Ag P.f/Pan (Standard Diagnostics Inc., Korea), RDT kits as outlined in the manufacturers’ instructions inserts.

#### Fluorescence in situ hybridization assays

The FISH assays were conducted on all the samples as per the manufacturer’s instructions supplied as part of the *P. falciparum*/*P. vivax* FISH combo and *Plasmodium* Genus FISH Kit (ID-FISH Technology Inc., Palo Alto, CA, USA). Briefly, thin smears were prepared from EDTA whole blood mixed with smear preparation reagent (SPR) (3 parts blood: 1 part SPR by volume). Each smear was prepared from 4 μl of the mixture, dried in the air and fixed in methanol. Hybridization buffer with probe mix (12 μl) was added to each fixed smear. The smear was covered with a cover slip and placed in a humid chamber at 37 °C for 30 min to allow for hybridization to occur. The smear was then washed with wash buffer twice, rinsed with rinse buffer and dried in the dark. A drop of *plasmodium* counterstain was added to the dry smear. The smear was then covered with a cover slip and examined using a fluorescence microscope (magnification: × 1000) fitted with a green filter (excitation and emission of 560 nm and 630 nm respectively).

#### Polymerase chain reaction

Briefly, PCR analyses were performed on purified DNA using primers (Forward 5′- GCTCTTTCTTGATTTCTTGGATG -3′ and Reverse 5’-AGCAGGTTAAGATCTCGTTCG-3′) for plasmodium genus using Rotor gene Q real time PCR cycler.

### Data management and statistical analysis

Data were entered in Microsoft Excel spread sheet and imported into Stata 13.0 for statistical analysis. Performance of the diagnostic methods was assessed with the combination of PCR and GM results serving as the gold standard for malaria detection. All but one sample which were positive by GM were also positive by PCR. The sample was taken as part of the reference standard. Demographic attributes of the study participants were summarized using appropriate descriptive statistics. The number of true positives (TP), true negatives (TN), false positives (FP) and false negatives (FN) were computed. Sensitivity was calculated as TP/ (TP + FN). Specificity was calculated as TN/ (TN + FP). The positive predictive value (PPV) was calculated as TP/(TP + FP) and negative predictive value (NPV) as TN/ (FN + TN). The accuracy of the test, defined as proportion of all tests that gave a correct result, was calculated as (TP + TN)/number of all tests. Variations in proportions of outcomes based on different diagnostic techniques were tested using chi square (χ2) test. For all statistical tests, the threshold for statistical significance was set at a *p*-value of less than 0.05.

The density of malaria parasite was determined as follows [28]:

$$ Number of parasites/\mu l\  blood=\frac{Number of parasites observed\ x\ 8000\ }{Number of white blood\kern0.5em cells observed\ } $$

Cohen’s κ was used to determine if there was agreement between FISH, Giemsa-microscopy, microscopy and/or PCR and RDT in the diagnoses of malaria.

## Results

### Demographic characteristics of the study participants

A total of 302 children were enrolled in the study with the majority being females (65.6%). Their ages ranged from one to 79 months with the median (interquartile range) age being 21 (9–33) months. Children less than 6 months of age constituted 18.2%; between 6 and 24 months, 40.4%; between 25 and 59 months, 39.7%; and >60 months, 1.7% of the study participants.

### Prevalence of malaria

The prevalence of infections with malaria parasites among the samples tested using the four diagnostic techniques under study is shown in Table 1. Overall, 81 (28.1%) and 104 (34.4%) samples were positive for malaria parasites when tested using FISH and RDTs, respectively. Eight nine samples (29.5%) were positive for malaria parasites based on PCR results only. Ninety samples (29.8%) were positive for malaria parasites by microscopy and/or PCR.

**Table 1:** Summary of *Plasmodium* Genus results based on diagnostic tests utilized in the study

Test	Positive		Negative	
	n	%	n	%
FISH	85	28.1	217	71.9
RDT	104	34.4	198	65.6
Microscopy	74	24.5	228	75.5
PCR	89	29.5	213	70.5
PCR &/or Microscopy	90	29.8	212	70.2

### Evaluation of the performance of the diagnostic tests

The sensitivity, specificity, positive predictive value (PPV), and negative predictive value (NPV) of the tests are shown in Table 2. In the present study, *Plasmodium* infections diagnosed using PCR and/or microscopy were regarded as the “true positives” with the rest being regarded as “true negatives”. The sensitivity and specificity of FISH was, 85.6% and 96.2% respectively, while that of microscopy was 82.2% and 100% respectively. RDT and PCR had sensitivities of, 91.1% and 98.9% respectively. The corresponding specificities were 89.6% and 100%.The findings on the predictive values of the tests are shown on Table 2b. The positive predictive values (PPV) for RDT, microscopy, FISH and PCR were 78.8%, 100%, 90.6% and 100% respectively. The negative predictive values (NPV) for RDT, microscopy, FISH and PCR were, 96.0%, 93.0%, 94.0% and 99.5% respectively.

**Table 2:** Comparison of Microscopy, FISH and RDT with Microscopy + PCR as Gold Standard

Test	% (95% Confidence limit)			
	Sensitivity	Specificity	Positive predictive value	Negative predictive value
RDT	91.1(83.4–95.4)	78.8 (70.0–85.6)	96.0 (92.2–97.9)	89.6 (84.8–93.1)
Microscopy	82.2 (73.1–88.8)	100.0 (95.1–100.0)	93.0 (88.9–95.6)	100.0 (98.2–100.0)
FISH	85.6 (76.8–91.4)	90.6 (82.5–95.2)	94.0 (90.0–96.5)	96.2 (92.7–98.1)
PCR	98.9 (94.0–99.8)	100.0 (95.9–100.0)	99.5 (97.4–99.9)	100.0 (98.2–100.0)

### Diagnostic effectiveness (accuracy)

Diagnostic accuracy was expressed as a sum of the proportion of correctly diagnosed samples (true positives (TP) and true negatives (TN)) out of all the samples tested. The overall diagnostic accuracy of FISH was 93.0% while that of microscopy was 94.7%. RDT and PCR had an accuracy of 90.1% and 99.7% respectively, (Table 3).

**Table 3:** Assessment of the diagnostic accuracy of FISH, RDT, GM and PCR in malaria diagnosis against a combination PCR and GM as the gold standard

Test	Accuracy	
	No. (*n* = 302)	% (95% CI)
RDT	272	90.1 (86.2–93.0)
Microscopy	286	94.7 (91.6–96.7)
FISH	281	93.0 (89.6–95.4)
PCR	301	99.7 (98.2–99.4)

#### Reliability - assessment of the agreement between the selected diagnostic approaches

There was high level of agreement in the results of a combination of Giemsa-microscopy and PCR when assessed against FISH (κ = 0.803, *p* < 0.001). Similarly, high levels of agreement in the results were observed when Giemsa-microscopy and PCR were assessed singly against FISH (κ = 0.804, *p* < 0.001 and κ = 0.822, *p* < 0.001, respectively). In addition, a moderate level of agreement was observed between the results obtained from RDT and those obtained by FISH (κ = 0.686, *p* < 0.001).

### Malaria parasite density

Analysis of the parasitaemia levels in the 74 samples (24.5%) that tested positive for malaria showed that the parasite levels ranged between 173 and 229,533 parasites/μL of blood. The geometric mean malaria parasite density was 15,631 parasites/μL of blood. The majority of samples (51, 71.6%) had high levels of parasitaemia (≥5000 parasites/μl of blood). The number of cases with low (1–999 parasites/μL of blood) and medium malaria parasite densities (1000–4999 parasites/μL of blood) were 14 (18.9%) and 7 (9.5%) respectively of the samples that were positive for malaria parasites.

## Discussion

The current study was undertaken to evaluate the fluorescence in situ hybridization (FISH) based technique for detecting *Plasmodium* sp. infection in blood smears when compared to Giemsa microscopy, PCR and RDTs. FISH is a potential test for use in malaria endemic resource-limited settings. In particular, the use of light emitting diode (LED) as a source of light makes the technique invaluable in such settings, which characterize many sub-Saharan African countries [21]. It has been demonstrated that the LED unit with a set of blue-green filters, attached to a regular light microscope with 100X objective can be used to read processed FISH smears [21]. Prior to the advent of LED units, FISH processed smears were read using mercury arc lamp fluorescence microscopes [29–31]. This made the technique expensive in terms of acquisition of microscopes and also the high cost of maintenance since mercury arc lamps have a relatively short lifespan. Furthermore, the disposal of the lamps raised concerns as mercury is considered a health hazard [21]. The present LED unit with attendant light filters is relatively cheaper and offers several advantages including; (a) it is mounted onto a regular microscope, (b) it is user-friendly, (c) it can operate on a rechargeable battery unit, thus usable in remote rural areas; (d) maintenance costs are minimal since a LED unit has a lifetime of more than 10,000 h (without decaying curve); (e) the LED light source, unlike mercury bulbs, requires no focus adjustments; and (f) the LED unit does not pose any health hazard. Apart from addressing this shortcoming, the FISH assays are also able to detect as well as differentiate between the various *Plasmodium* species. The FISH assays detected all stages of the malaria parasites present in blood including gametocytes [21].

The potential of FISH as a vital tool for monitoring the efficacy of treatment for malaria is being considered. This is based on the fact that it detects only live parasites. FISH detects specific 18S rRNA fragments in live parasites whereas PCR, RDT and Giemsa microscopy do not distinguish between live and dead parasites. In case of microscopy, parasite detection and speciation is based on morphology only [28], whereas FISH, in addition to speciation, based on presence of specific 18S rRNA sequences, provides morphological information [21, 32].Therefore, the potential use of FISH in diagnosis, as part of the national testing algorithm, may potentially lead to the avoidance of unnecessary treatments which ultimately will enable the National Malaria Control Programme to have a better handle on the development of resistance to antimalarials [33].

In the study, it was noted that the FISH assay had the higher sensitivity (86%) when compared to the Giemsa-microscopy (82%) diagnostic approach which is routinely used in diagnosis centres across the country. On the other hand, it had lower sensitivity when evaluated against the RDT (91%). As expected, PCR registered the highest sensitivity as compared to all other diagnostic approaches (99%). The findings in this study corroborates those of a similar study by Shah and others who reported that FISH assays were more sensitive than GM. Shah and her team found that the sensitivities of P-Genus, PF and PV FISH assays to be 98.2%, 94.5% and 98.3%, respectively compared to 89.9%, 83.3% and 87.9% for the detection of *P. falciparum* and *P. vivax* by GM respectively [21]. In the present study, 83% (9/11) of the samples between 280 and 1000 parasites/μl of blood and 98% (59/60) samples with >2000 parasites/μl blood were detected by FISH. Also According to Shah et al., the limit of detection for Plasmodium Genus FISH is 170 parasites/μl blood [21]. Therefore, it is interesting that Osoga et al., only detected 42% (22/52) of the samples with >2000 parasites/μl blood and 90% of samples with 2 parasites/μl blood [23].

In the current study, specificities were highest in both PCR and GM (100%), whereas RDT showed the lowest specificity (90%). The specificity of FISH assay (96%) was hence comparable to that of PCR and GM. Another study reported a much lower specificity for FISH assays and RDT (88.2% and 97.6% respectively) [21]. The discrepancies in the findings from the two studies may be attributed to, at least in part, the fact that the latter study used GM as a reference method while our study relied on PCR results in addition to Giemsa microscopy. Other studies have equally shown the superiority of FISH technique as a diagnostic tool for studying pathogens other than malaria parasites. Indeed, when 22 pathogen smears were tested plasmodium by FISH assays, no cross-reaction was observed. The pathogens included 4 parasites (*Trypanosoma cruzi*, *Babesia duncani*, *B. microti and Leishmania major*), 7 bacteria and 11 viruses [34]. Similarly, Sharon and others evaluated the potential utilization of FISH for the detection and differentiation of tuberculosis from *Mycobacterium tuberculosis* infections in resource-constrained settings. They established that sensitivities for the *Mycobacterium* spp. and *Mycobacterium tuberculosis* FISH assay of sputum were 82% and 89%; and specificities were 91% and 95%, respectively [35].

In this study, the diagnostic accuracy of FISH assay was 93.0%, which was higher than that of RDTs (90%) though not statistically significant. Diagnostic accuracy of FISH assay was comparable to that of microscopy which was 95%. Although PCR had diagnostic accuracy of about 100% its utility in malaria endemic areas is limited by the complexity of the requisite methodologies in addition to the high costs, in terms of the facilities required and the advanced level of staff training needed. Moreover, maintenance of PCR equipment is also critical, further lowering its suitability for diagnosis of malaria in remote settings and in routine clinical work [16]. On the contrary, the FISH technique is comparatively inexpensive, as it does not require high maintenance equipment. Notably, the reagents used in FISH assays are relatively thermal-stable which makes the technology useful in rural area settings.

On evaluation of the agreement between the various diagnostic approaches studied, FISH assays showed a high level of reliability in the diagnoses of malaria with the agreement with Giemsa-microscopy, and also microscopy and/or PCR being rated as high. Moderate level of agreement was found between FISH and RDT. In their research, Shah et al. also demonstrated the reproducibility of the FISH assays [21].

The present study has a number of limitations. The study did not assess the variations in the performance of the FISH assays based on the levels of parasitaemia in blood (parasite densities). Considering that FISH assays detect only live parasites while other diagnostic approaches including Giemsa microscopy detect both live and dead parasites there may be a difference in performance of the various malaria diagnostic tools [33]. The study also did not disaggregate the performance of FISH assay according to the different species of malaria parasites. However, the study provides important evidence that FISH technique may be a reasonable assay to use especially in resource-limited settings where there is a need for a sensitive, affordable and technologically sound tool for timely diagnosis of malaria.

## Conclusion

This study highlights the potential use of FISH assay as an effective diagnostic tool for the detection of malaria parasites in resource-constrained settings. The specificity and reproducibility of FISH assay were found to be high. The adoption of this assay may well promote better clinical practices such as monitoring the efficacy of malaria treatment in addition to avoiding unnecessary, or minimizing empirical treatments, as it detects live parasites only. This, in turn, will lower the risk of development of drug resistance, ultimately resulting in substantial cost savings from avoidance of unwarranted treatments of patients. There remains a need to conduct further similar studies in a field setup and in different malaria epidemiological zones so as to further assess the effectiveness of this diagnostic tool. The performance of the tool also needs to be evaluated against various *Plasmodium* species as well as in the mixed infections cases.

## Abbreviations

FISH: Fluorescence in situ hybridization
FN: False negatives
FP: False positives
GM: Giemsa microscopy
GOK: Government of Kenya
LED: Light emitting diode
MOPHS: Ministry of Public Health and Sanitation
NPV: Negative predictive value
PCR: Polymerase chain reaction
PPV: Positive predictive value
RDT: Rapid diagnostic test
TN: True negatives
TP: True positives
Unicef: United Nations Children Fund
WHO: World Health Organization
